# Performance of Foamed Concrete Incorporating Modified Expanded Perlite

**DOI:** 10.3390/ma19204350

**Published:** 2026-10-16

**Authors:** Xun Xu, Haiyan Sun, Peng Zhou, Xing Kang, Yuchang Gao

**Affiliations:** College of Water Conservancy, Yunnan Agricultural University, Kunming 650201, China; 15334430780@163.com (X.X.); 15126612162@163.com (P.Z.); 19187414984@163.com (X.K.); 15198772823@163.com (Y.G.)

**Keywords:** foamed concrete, expanded perlite, hydrophobic modification, compressive strength, water absorption, pore structure

## Abstract

This study investigates the influence of hydrophobically modified expanded perlite on the properties of foamed concrete. Expanded perlite treated with 6 wt% sodium methylsilicate was adopted as lightweight aggregate at replacement levels of 0 wt%, 5 wt%, 10 wt%, 15 wt% and 20 wt%. The physical, mechanical and durability properties including dry density, water absorption, compressive strength, drying shrinkage and freeze–thaw resistance were systematically tested. Optical microscopy and scanning electron microscopy (SEM) were further used to characterize pore characteristics and hydration products, so as to reveal the mechanisms governing the macroscopic performance of foamed concrete. The results indicate that an appropriate dosage of hydrophobically modified expanded perlite can increase the compressive strength of foamed concrete and reduce its 48 h water absorption. When the replacement dosage of modified expanded perlite reaches 10 wt%, the compressive strength reaches 7.15 MPa, which is 111% higher than that of the 0 wt% group, while the 48 h water absorption decreases from 20.18% to 8.74%. Nevertheless, drying shrinkage increases continuously with the increase in modified expanded perlite dosage. Proper addition of modified expanded perlite refines pore structure and improves the freeze–thaw resistance of foamed concrete. The mixture with 10 wt% modified expanded perlite presents relatively balanced performance considering compressive strength and frost resistance. This work provides experimental basis and technical support for the engineering application of perlite-based lightweight foamed concrete.

## 1. Introduction

Driven by the global energy crisis and the carbon peaking and carbon neutrality goals, the construction industry, as a major energy-consuming sector, faces increasingly urgent demands for energy conservation and emission reduction. Statistics show that building energy consumption accounts for approximately 40% of total social energy use in China [1,2]. Heating and cooling consume the largest share, and heat loss through walls is one of the main pathways of building energy loss. Therefore, the development of new wall materials that combine low density, high strength, and excellent thermal insulation has become a key route toward reducing building energy consumption and promoting the transition to green construction [3].

Foamed concrete is a lightweight porous cement-based material containing a large number of air voids [4]. Compared with ordinary concrete, it has the advantages of low density, high porosity [5,6], and excellent sound insulation, thermal insulation, and fire resistance. It has been widely used in roof and wall insulation layers, backfilling works [7,8], lightweight masonry blocks, and road and bridge engineering. Expanded perlite is also widely used in the building materials industry because of its low density, good thermal insulation, and low cost. In particular, it can be introduced into foamed concrete as a lightweight aggregate for the preparation of thermal insulation mortars, fire-resistant door cores, insulation panels, and other insulating products. In addition, lightweight aggregate concrete prepared with expanded perlite offers good wear resistance, thermal insulation, dimensional stability, and no alkali–aggregate reaction risk. Compared with ordinary concrete of the same strength grade, its self-weight can be reduced by 20–25%, giving it remarkable application advantages [9,10]. Numerous studies have investigated the effect of expanded perlite on foamed concrete properties. Although the conclusions are not entirely consistent, they all confirm that expanded perlite exerts a significant regulatory effect on the performance of foamed concrete.

Previous research has shown that adding an appropriate amount of expanded perlite can effectively improve the strength of foamed concrete, with 28-day compressive strength reaching 3.2 MPa in some cases [11]. Similar trends have also been observed in slag-based foamed concrete containing expanded perlite and glazed hollow beads, where compressive strength first increases and then decreases with increasing perlite content, reaching a peak at 3% [12]. Studies by other authors have likewise demonstrated that a moderate dosage of expanded perlite benefits the mechanical properties of mortar and lightweight concrete [13,14]. However, due to the highly porous structure of expanded perlite, water absorption tends to increase substantially as perlite content rises [15,16]. In foamed concrete made with fly ash and expanded perlite, increasing the perlite content led to a continuous decrease in compressive strength and a corresponding increase in water absorption. Compared with the control group, a 20% dosage reduced compressive strength by 8.7% and increased water absorption by 27.8% [17].

In summary, expanded perlite has been widely studied as a lightweight aggregate for foamed concrete because of its low density, thermal insulation, and low cost. It is commonly used to optimize bulk density, mechanical properties, and insulation performance. However, its intrinsically porous structure causes strong water absorption, which can significantly increase the overall water absorption of foamed concrete after direct incorporation. This in turn weakens the interfacial bonding of the matrix and deteriorates frost resistance, thereby severely limiting engineering application. Most existing studies adopt untreated raw expanded perlite, while limited literature is available on foamed concrete incorporating hydrophobically modified expanded perlite, particularly regarding the multi-scale evolution of pore structure and the underlying hydrophobization mechanism.

To address the above-mentioned problem, this study first modified expanded perlite with sodium methylsilicate to obtain hydrophobic expanded perlite. Foamed-concrete mixtures were prepared with modified expanded perlite replacement ranging from 0 wt% to 20 wt% at 5 wt% intervals. The physical, mechanical, and durability properties of foamed concrete were systematically evaluated. Combined with pore structure and microstructure characterization, the underlying working mechanism was revealed. The findings are expected to provide a theoretical basis and technical support for the engineering application of foamed concrete containing expanded perlite.

## 2. Materials and Methods

### 2.1. Raw Materials

Cement: P.O 42.5 ordinary Portland cement produced by Yunnan Huaxin Dongjun Cement Co., Ltd. (Kunming, China); its main physical properties are listed in Table 1. Expanded perlite: supplied by Hebei Yixin Energy-Saving Insulation Building Materials Co., Ltd. (Langfan, China), with a particle size of 2–4 mm and a bulk density of 100 kg/m^3^. Foaming agent: animal-protein-based foaming agent produced by Jiangsu Ting’an Fire Protection Materials Co., Ltd. (Taizhou, China). Foam stabilizer: calcium stearate, produced by Tianjin Huasheng Chemical Reagent Co., Ltd. (Tianjin, China). Hydrophobic modifier: sodium methylsilicate, produced by Beijing Mongtai Weiye Building Materials Co., Ltd. (Beijing, China). Mixing water: ordinary tap water.

The water absorption of raw expanded perlite is 412.5%, which decreases to 82% after hydrophobic modification using 6 wt% sodium methylsilicate solution, confirming the effectiveness of the hydrophobic treatment. Contact angle measurement was not performed in the present work; relevant surface characterization will be supplemented in future investigations. The modification parameters (6 wt% sodium methylsilicate solution, 2 h immersion, and drying at 60 °C for 48 h) were selected according to relevant literature [18] combined with our preliminary tests.

### 2.2. Mix Design

In this study, modified expanded perlite was used to replace cement on an equal-mass basis. That is, the mass of reduced cement is equal to the added mass of modified expanded perlite. Owing to the very low bulk density of expanded perlite, this equal-mass replacement brings considerable changes to the solid-phase volume fraction of mixtures.

The target dry density of the foamed concrete was designed to be 800 kg/m^3^. The water-to-cement ratio was 0.4, and the dosage of foam stabilizer was 0.6% of the cement mass. A foam surplus factor of approximately 1.10 was adopted to compensate for expected foam loss during mixing and casting, following the mix design method of Li et al. [19].

The mix design was based on the absolute-volume method. The apparent density of modified expanded perlite was approximately 250 kg/m^3^ (based on supplier data and preliminary measurements); note that this differs from the bulk density (100 kg/m^3^) reported in Section 2.1, as the bulk density includes inter-particle voids. The other material densities used were: cement = 3100 kg/m^3^, water = 1000 kg/m^3^, and foam = 60 kg/m^3^. The absolute-volume calculation results for each mixture are summarized in Table 2.

### 2.3. Specimen Preparation

First, sodium methylsilicate hydrophobic agent was prepared as an aqueous solution with a mass fraction of 6%. An appropriate amount of expanded perlite was then immersed in the solution for 2 h, followed by drying in an oven at 60 °C for 48 h until constant mass was reached. The treated material was sealed for later use, yielding modified expanded perlite.

**Figure 1:**
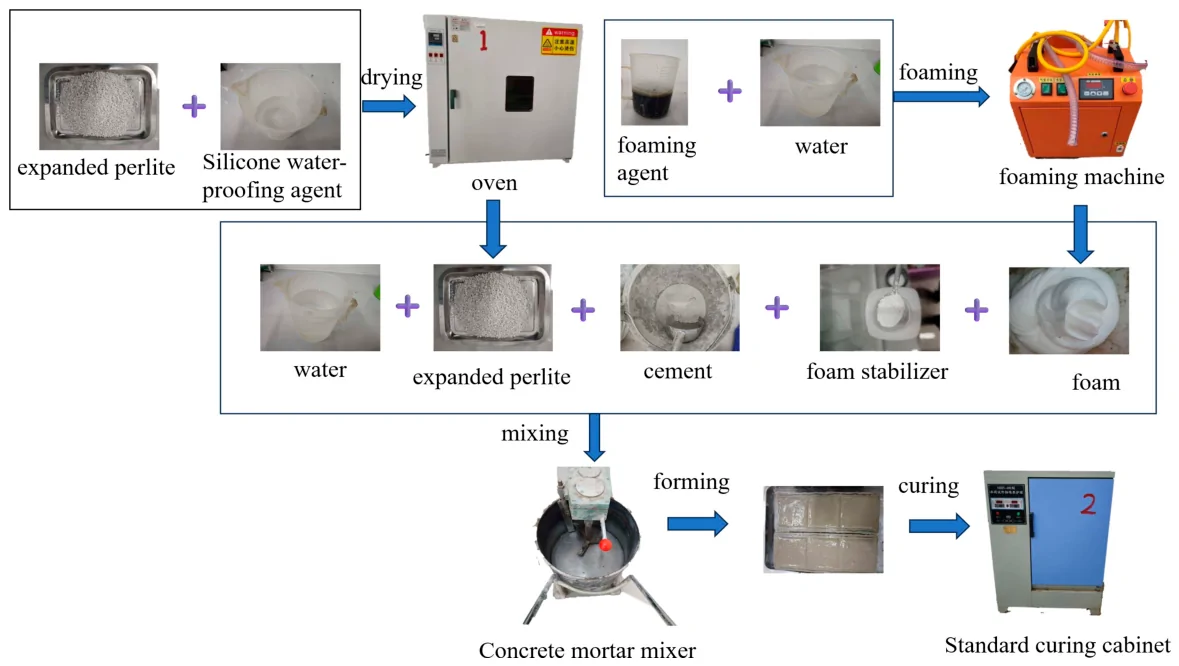
Preparation route for foamed concrete containing modified expanded perlite.

The foamed concrete preparation process is shown schematically in Figure 1. The materials were weighed according to the mix proportions described in Section 2.2. Cement, foam stabilizer, and modified expanded perlite were slowly poured into an SJ-15 mortar mixer and dry-mixed for 2 min. Two-thirds of the water was then added and mixed for 2 min, followed by the remaining one-third of the water and mixing for 3 min to obtain a uniform cement-based slurry, which was then set aside. Next, the foaming agent was diluted with water at a ratio of 1:40, and foam was generated using a foaming machine. The required amount of foam was weighed and added to the slurry within 1 min. A two-step foaming method was adopted: 20–30% of the foam was first added to facilitate dispersion, after which the remaining foam was introduced. The mixture was then stirred for another 2 min, left to stand for 15 s to remove foam adhering to the inner wall, and low-speed mixed again for 1 min. The fresh mixture was cast into the corresponding molds, covered with film, and cured at room temperature for 48 h. The specimens were then demolded and cured in a standard curing chamber for 28 d before testing.

### 2.4. Test Methods

For each mixture and each performance index, three replicate specimens were generally prepared. Individual groups had only two valid replicates because some specimens were damaged during testing.

The dry density and compressive strength of foamed concrete were tested according to JG/T 266-2011 [20] Foamed Concrete, using specimens of 100 mm × 100 mm × 100 mm. The water-absorption immersion test adopted in this manuscript follows self-defined immersion time intervals and differs from the test procedure specified in JG/T 266-2011.

The frost-resistance test was carried out following the rapid freeze–thaw method specified in GB/T 50082-2009 [21], with specimen dimensions of 100 mm × 100 mm × 400 mm. Mass loss ratio and relative dynamic modulus of elasticity were recorded every 25 freeze–thaw cycles.

The drying-shrinkage test was performed in accordance with GB/T 50082-2009, and the gauge length for shrinkage measurement was 515 mm.

**Figure 2:**
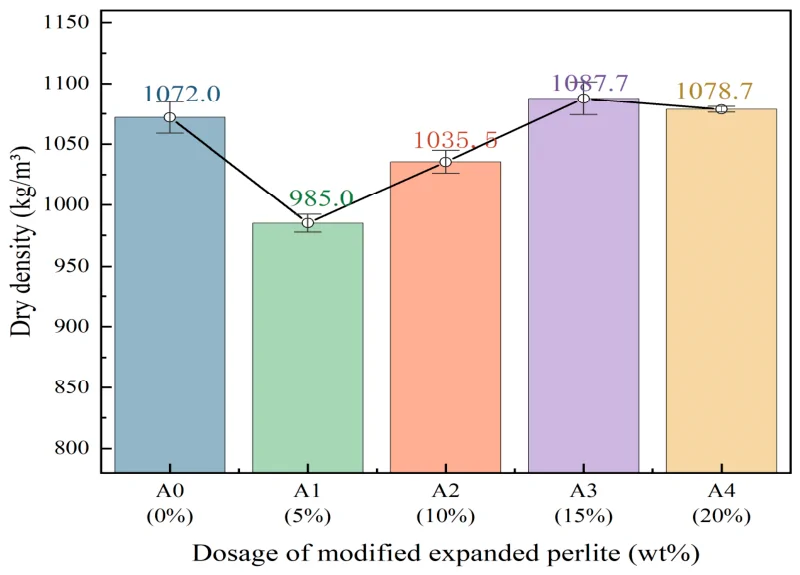
Dry density of foamed concrete with different contents of modified expanded perlite.

**Figure 3:**
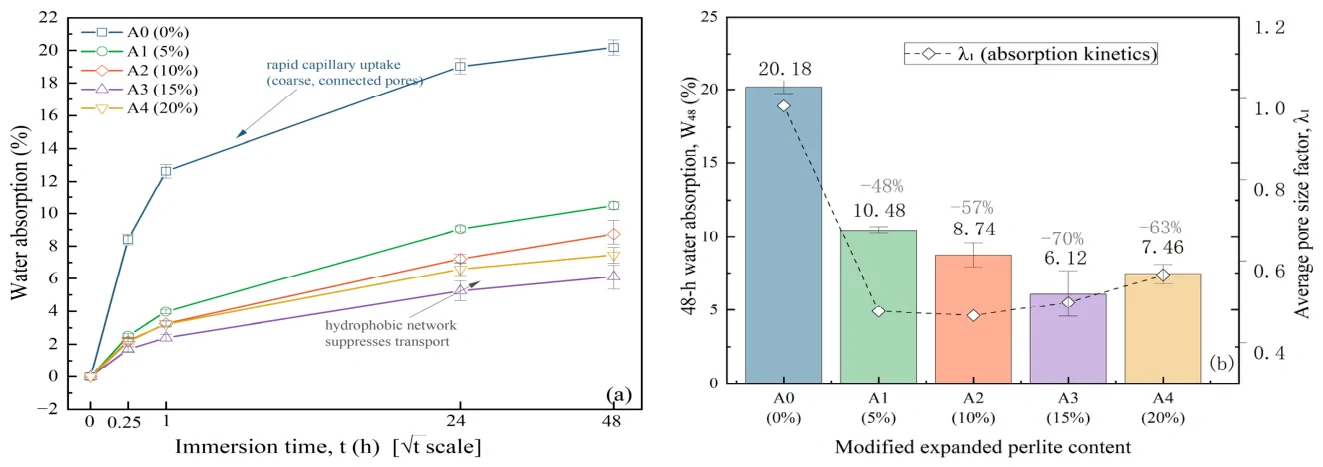
Water-absorption behavior of foamed concrete: (**a**) water absorption versus immersion time (plotted against $\sqrt{t}$); (**b**) 48 h water absorption and average pore-size factor λ_1_.

**Figure 4:**
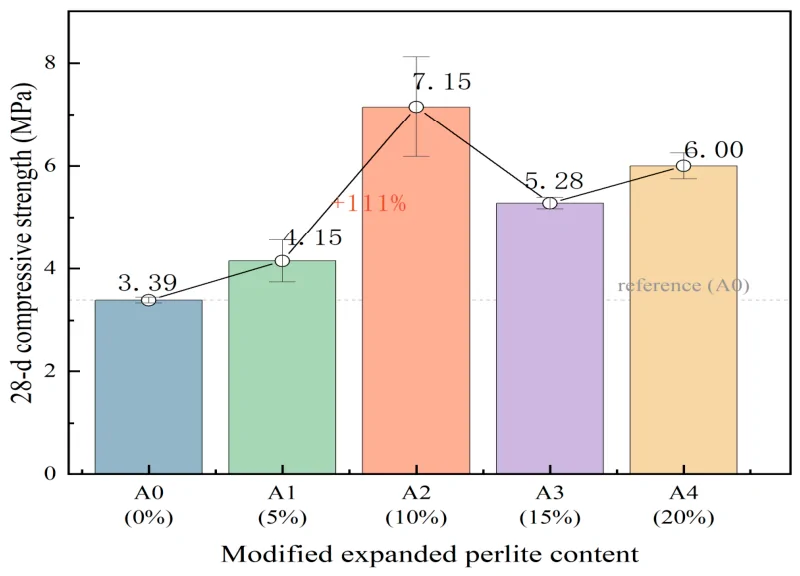
Compressive strength of foamed concrete with different contents of modified expanded perlite.

For each mixture and each performance index, three replicate specimens were prepared and tested, and the results are expressed as mean ± standard deviation (SD). Error bars representing SD are included in Figure 2, Figure 3b and Figure 4. For pore-size analysis, two optical-microscopy images were acquired per mixture, and more than 80 pores were counted by ImageJ software version 1.54g (National Institutes of Health, Bethesda, MD, USA).

The following equipment was used for testing: a universal testing machine (Shenzhen Wance Testing Machine Co., Ltd., Shenzhen, China), a rapid freeze–thaw chamber (Beijing Shuzhi Yilong Instrument Co., Ltd., Beijing, China), a DT-W18 dynamic elastic modulus tester (Beijing Aerospace Huayu Test Instrument Co., Ltd., Beijing, China), an optical microscope (Carl Zeiss, Oberkochen, Germany), and a DB500 focused ion beam scanning electron microscope (Guoyi Quantum Technology Co., Ltd., Hefei, China).

**Figure 5:**
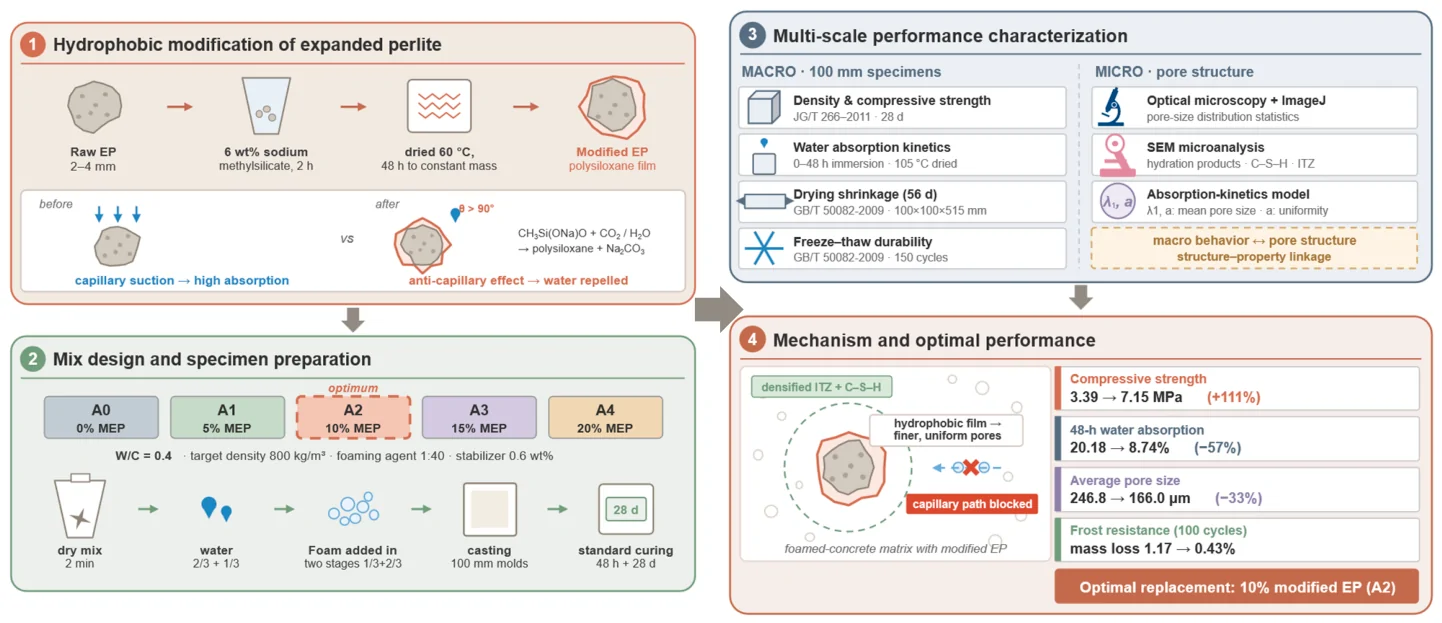
Research framework of this study: (**1**) hydrophobic modification of expanded perlite (EP) with sodium methylsilicate; (**2**) mix design and two-step foaming specimen preparation; (**3**) multi-scale performance characterization; (**4**) mechanism analysis and identification of the optimal replacement level.

The overall research framework of this study is summarized in Figure 5. Hydrophobic modification converts the strongly absorbent raw expanded perlite into a water-repellent lightweight aggregate through the in situ formation of a polysiloxane film. The modified aggregate is then introduced into foamed concrete at five replacement levels (A0–A4), and the resulting mixtures are evaluated by a multi-scale characterization program that couples macroscopic performance tests with pore structure and microstructural analyses, so that the macroscopic property evolution can be traced back to its underlying hydrophobization and pore-refinement mechanisms.

## 3. Results and Discussion

### 3.1. Dry Density

The influence of modified expanded perlite content on the dry density of foamed concrete is shown in Figure 2. As the dosage increased from 0% to 20%, the dry density initially decreased and then increased. When the dosage increased from 0% to 5%, the dry density decreased from 1072.0 kg/m^3^ to 985.0 kg/m^3^, representing a reduction of 8.1%. At low dosages, the density of hydrophobically modified perlite is much lower than that of cement. The incorporation of the lightweight perlite dilutes the cement proportion, thereby reducing the dry density. When the dosage increased further to 10%, 15%, and 20%, the dry density rose to 1035.5 kg/m^3^, 1087.7 kg/m^3^, and 1078.7 kg/m^3^, respectively. This is attributed to the hydrophobic film formed on the perlite surface after modification, which reduces water absorption from the slurry and prevents bubble rupture caused by water loss. As a result, the foam structure becomes more stable, the bubble size distribution becomes more uniform, and the density of the specimens increases. When the dosage exceeded 15%, the large amount of low-density modified perlite further diluted the cement content. Meanwhile, excessive aggregate led to insufficient slurry encapsulation and increased internal porosity, which reduced compactness and caused the density to decline slightly.

The measured dry densities were generally higher than the design value. This is mainly because bubble rupture and dissipation occurred during mixing and forming, which substantially reduced the internal porosity and increased the overall compactness. In addition, sodium methylsilicate modification fills and blocks the micro-pores and cracks on the perlite surface, increasing the apparent density of the aggregate and weakening the weight-reduction effect. Mechanical shearing during mixing may also break modified perlite particles into fine powder, and these fine particles fill the gaps and continuously compress the pore space, further increasing density. At low dosages, modified expanded perlite slightly optimizes the density of the system; however, at higher dosages, bubble loss, aggregate densification, and powder filling dominate, offsetting the lightweight effect and causing the dry density to rebound.

Furthermore, the theoretical fresh density calculated from the absolute-volume method (Table 2) ranges from 616.3 to 923.8 kg/m^3^, which is substantially lower than the measured dry density (985.0–1087.7 kg/m^3^). Although a foam surplus factor of approximately 1.10 was adopted during mix design to compensate for expected foam loss [19], the actual foam retention was far lower than anticipated; only about 25% of the designed foam volume was retained in the hardened specimens (estimated by comparing the theoretical fresh volume with the actual dry volume). This severe foam loss during mixing and casting is the primary reason why the measured dry densities substantially exceed both the target value (800 kg/m^3^) and the theoretical fresh density. Notably, even the reference group A0 without perlite shows a measured dry density far above the target value of 800 kg/m^3^, quantitatively demonstrating that bubble damage during mixing and casting is the primary source of this deviation.

### 3.2. Water Absorption

To obtain water-absorption and pore characteristic parameters simultaneously, an absorption-kinetics method was used in this study. The specimens were dried at 105 °C for 48 h to constant mass, cooled to room temperature, and then fully immersed in water. Their mass changes were recorded at 0 h, 0.25 h, 1 h, 24 h, and 48 h. Under isothermal conditions, foamed concrete, as a typical porous medium, exhibits a stable exponential water-absorption behavior [22,23]. On this basis, the pore structure was quantitatively characterized using the water-absorption-kinetics method. The applied kinetic model is shown in Equation (1):

(1) $$w_{t}=w_{\max}(1-e^{-\lambda_{1}t^{\alpha }})$$

where

*W_t_* = water-absorption ratio at immersion time *t* (%);

*W_max_* = saturated water-absorption ratio (%);

*λ*_1_ = pore-related absorption coefficient (h^−α^);

*α* = pore-size uniformity coefficient (dimensionless, ranging 0–1; a smaller value indicates poorer pore-size uniformity);

*t* = immersion time (h). Table 3 lists the pore characteristic parameters calculated from the water-absorption-kinetics method. It can be observed that λ_1_ decreases from A0 to A2 and rises again from A2 to A4, while water absorption rebounds for group A4. Excessive addition of modified expanded perlite introduces more weak interfacial transition zones inside the matrix and regenerates partially connected water-transport channels, which accounts for this phenomenon.

Figure 3a shows the variation in water absorption with time for foamed concrete containing different dosages of modified expanded perlite, and Figure 3b compares the 48 h water absorption together with the average pore-size factor λ_1_. Specimens with lower modified perlite content exhibited a higher early-stage water absorption rate and overall water absorption, indicating a greater proportion of large pores. The water-absorption rate is consistent with the average pore-size analysis shown in Table 3: the larger the average pore size, the faster the capillary absorption at the early stage. As the dosage of modified expanded perlite increased, water absorption gradually decreased. When the dosage increased from 0% to 5%, 10%, 15%, and 20%, water absorption dropped from 20.18% to 10.49%, 8.74%, 6.11%, and 7.46%, respectively.

It is worth noting that, in contrast to the substantial increase in water absorption commonly observed when unmodified expanded perlite is incorporated into cement-based materials [15,16], this reduction is mainly attributed to the sodium methylsilicate hydrophobic modification of expanded perlite, which converts the highly absorbent raw perlite into a water-repellent lightweight aggregate. The modifier can penetrate the internal pores of perlite and react with atmospheric CO_2_ and H_2_O to form polysiloxane and sodium carbonate. The polysiloxane forms a continuous and dense hydrophobic film on the particle surface and pore walls, giving the surface a low-energy characteristic, while sodium carbonate fills part of the fine connected pores [18,24]. The hydrophobic film weakens the capillary driving force through the anti-capillary effect, thereby significantly enhancing the hydrophobicity of expanded perlite [25,26]. At low dosages, the foamed concrete matrix is still dominated by hydrophilic cementitious material and connected pores, resulting in higher water absorption. With increasing dosage, the number of hydrophobic particles increases, the effective space for storing water decreases, and a continuous hydrophobic network is gradually formed, which blocks connected channels for water transport and significantly reduces water absorption.

### 3.3. Compressive Strength

Figure 4 presents the compressive strength of foamed concrete with different contents of modified expanded perlite. The compressive strength first increased and then decreased with increasing dosage. When the dosage increased from 0% to 10%, the compressive strength rose from 3.39 MPa to 7.15 MPa, an increase of 111%, which is substantially higher than the 3.2 MPa reported for foamed concrete containing unmodified expanded perlite [11]. On the one hand, a moderate amount of modified expanded perlite is uniformly dispersed in the slurry and forms a composite skeleton with the cementitious matrix, enabling it to share the external load. The reasonable distribution of particles also optimizes stress transfer and suppresses the initiation and propagation of microcracks, thereby enhancing load-bearing capacity [27,28]. On the other hand, the hydrophobic modification of expanded perlite greatly reduces its water absorption, stabilizes the water-to-binder ratio, and ensures sufficient water for cement hydration, which provides a foundation for strength gain. Meanwhile, the dense hydrophobic film formed on the particle surface prevents excessive adsorption of pore solution, avoids the formation of weak and porous interfacial zones with an elevated local water-to-binder ratio and Ca(OH)_2_ enrichment, and improves bonding between aggregate and cement paste. This leads to a more stable interfacial structure and higher interfacial strength.

When the dosage increased from 10% to 15%, compressive strength declined. This is because the excessive volume fraction of aggregate reduces the proportion of the cementitious matrix, leading to insufficient hydration products and weaker bonding between aggregate and paste. In addition, during mixing and hardening, the bubble size inside the specimens increases, connected pores become more numerous, and the pore walls become thinner, resulting in more pronounced structural defects and a clear reduction in compressive strength [29,30]. When the dosage further increased from 15% to 20%, a densely packed and interlocked structure formed among the modified perlite particles, establishing a stable rigid aggregate skeleton, and the compressive strength increased again.

### 3.4. Drying Shrinkage

**Figure 6:**
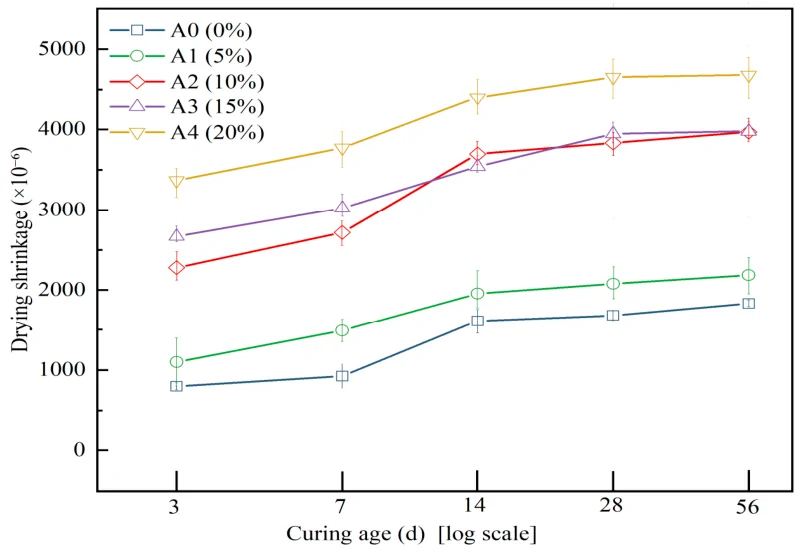
Drying shrinkage of foamed concrete at different curing ages.

Figure 6 shows the effect of modified expanded perlite content on the drying shrinkage of foamed concrete. It can be seen that drying shrinkage increased gradually as the perlite dosage increased. At an age of 56 d, compared with the A0 control mixture without perlite, the drying shrinkage of A1, A2, A3, and A4 increased by 19.3%, 116.6%, 117.0%, and 155.7%, respectively.

The increase in drying shrinkage with modified expanded perlite content is attributed to two main reasons. First, the hydrophobic film formed on the perlite surface reduces water absorption, preventing the aggregate from slowly releasing water during cement hydration to mitigate drying shrinkage. Second, as the modified perlite content rises, excessive particles disturb the homogeneity of the slurry setting, reduce the compactness of the matrix, and weaken particle bonding and shrinkage restraint. In addition, differences in swelling–shrinkage behavior and thermal expansion coefficients between the aggregate and the cementitious materials exacerbate internal stress imbalance, thereby amplifying drying shrinkage.

Detailed original drying-shrinkage numerical values at different curing ages are fully listed in Table 4 for cross-check with percentage-change descriptions within the text.

### 3.5. Frost Resistance

**Figure 7:**
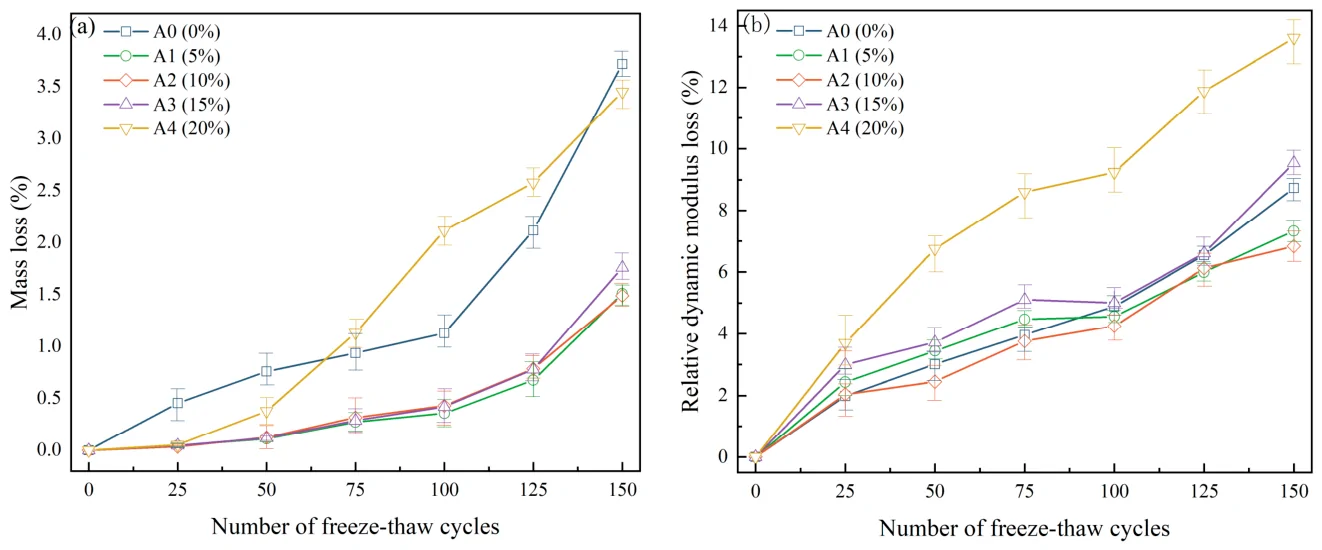
Freeze–thaw resistance of foamed concrete: (**a**) mass loss; (**b**) relative dynamic modulus of elasticity loss.

Figure 7a shows the variation in mass loss ratio with the number of freeze–thaw cycles. The specimens were subjected to 150 freeze–thaw cycles, and their mass was measured every 25 cycles. As the number of cycles increased, the mass loss ratio gradually increased. Taking mixture A2 with 10% modified expanded perlite as an example, the mass loss ratio increased from 0.034% after 25 cycles to 1.409% after 150 cycles, representing an increase of more than 40 times. This is due to the phase change of water inside the specimen during freezing and thawing: when the temperature drops, water in the pores freezes and expands, causing microcracks in the pore walls. Repeated freezing and thawing continuously expands these cracks and even connects them, ultimately leading to surface scaling and spalling, which results in mass loss [31].

At the same number of freeze–thaw cycles, the mass loss ratio generally decreased first and then increased with increasing modified expanded perlite dosage. At 100 freeze–thaw cycles, as the dosage increased from 0% to 5%, 10%, 15%, and 20%, the mass loss ratio decreased from 1.17% to 0.355%, 0.434%, and 0.42%, and then increased to 2.11%. These results indicate that an appropriate amount of modified expanded perlite can reduce water absorption, block the transmission of frost-heave stress, refine the internal pore structure, and mitigate freeze–thaw damage, thereby significantly lowering mass loss. However, at excessive dosage, too many lightweight aggregate particles weaken the overall matrix structure, increase the number and proportion of weak interfacial transition zones, and reduce the effective binding capacity of the cementitious material, causing internal defects to grow further under freeze–thaw action and leading to a pronounced increase in mass loss, consistent with the pore-refinement mechanism reported in previous frost-resistant concrete studies [31].

Figure 7b shows the variation in relative dynamic modulus of elasticity during freeze–thaw cycling. The trend is basically consistent with the mass loss ratio in Figure 7a. When an appropriate amount of modified expanded perlite is added, the hydrophobic film on the particle surface effectively reduces the amount of freezable water in the specimen, optimizes the pore structure, refines the pore size, and reduces the damage caused by freeze–thaw cycles, thereby improving frost resistance. When the dosage is too high, the overall mechanical strength of the specimen decreases and more weak interfacial transition zones appear. Under freeze–thaw stress, internal microcracks can initiate and propagate rapidly, leading to a significant reduction in relative dynamic modulus and a marked deterioration in frost resistance.

### 3.6. Pore Structure

**Figure 8:**
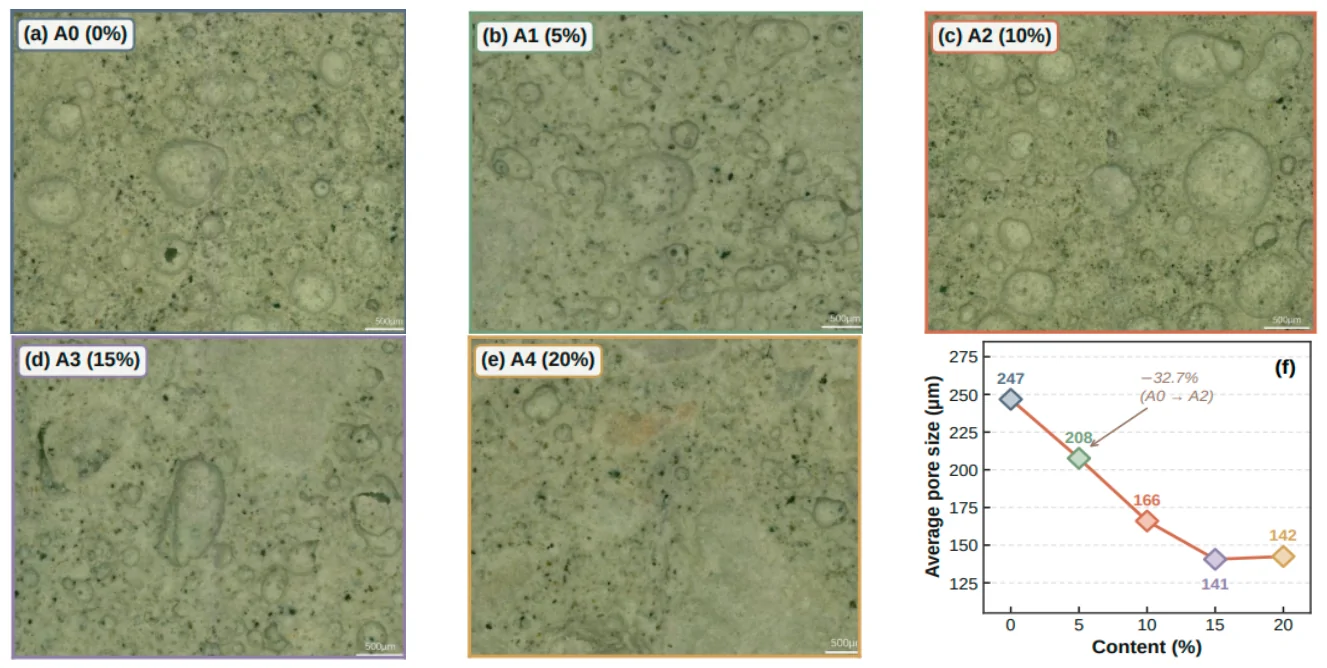
Pore structure of foamed concrete under optical microscopy at modified expanded perlite contents of (**a**) 0%, (**b**) 5%, (**c**) 10%, (**d**) 15%, and (**e**) 20%; (**f**) corresponding average pore size obtained from ImageJ analysis.

Figure 8 shows the pore morphology of foamed concrete observed by optical microscopy at different modified expanded perlite dosages. Optical microscopy images were acquired at a magnification of 50×, and a scale bar is included in each image. It is evident that the incorporation of modified expanded perlite significantly regulates the internal pore structure of foamed concrete. As the dosage increases, the average pore size gradually decreases and the number of large pores decreases accordingly.

When the dosage was 0% (Figure 8a), the pore size of the control specimen was relatively large, with a high proportion of large-diameter pores and an uneven pore-size distribution. After the dosage increased to 5% (Figure 8b), the pore size became smaller than that of the control group, the proportion of large pores decreased, the number of medium-sized pores increased, and pore uniformity improved. At 10% (Figure 8c), the pore size decreased further, leaving only a small number of large pores, and the structure was dominated by medium and small pores. When the dosage increased to 15% (Figure 8d) and 20% (Figure 8e), the pore size was further refined, and the surface was dominated by small pores.

**Figure 9:**
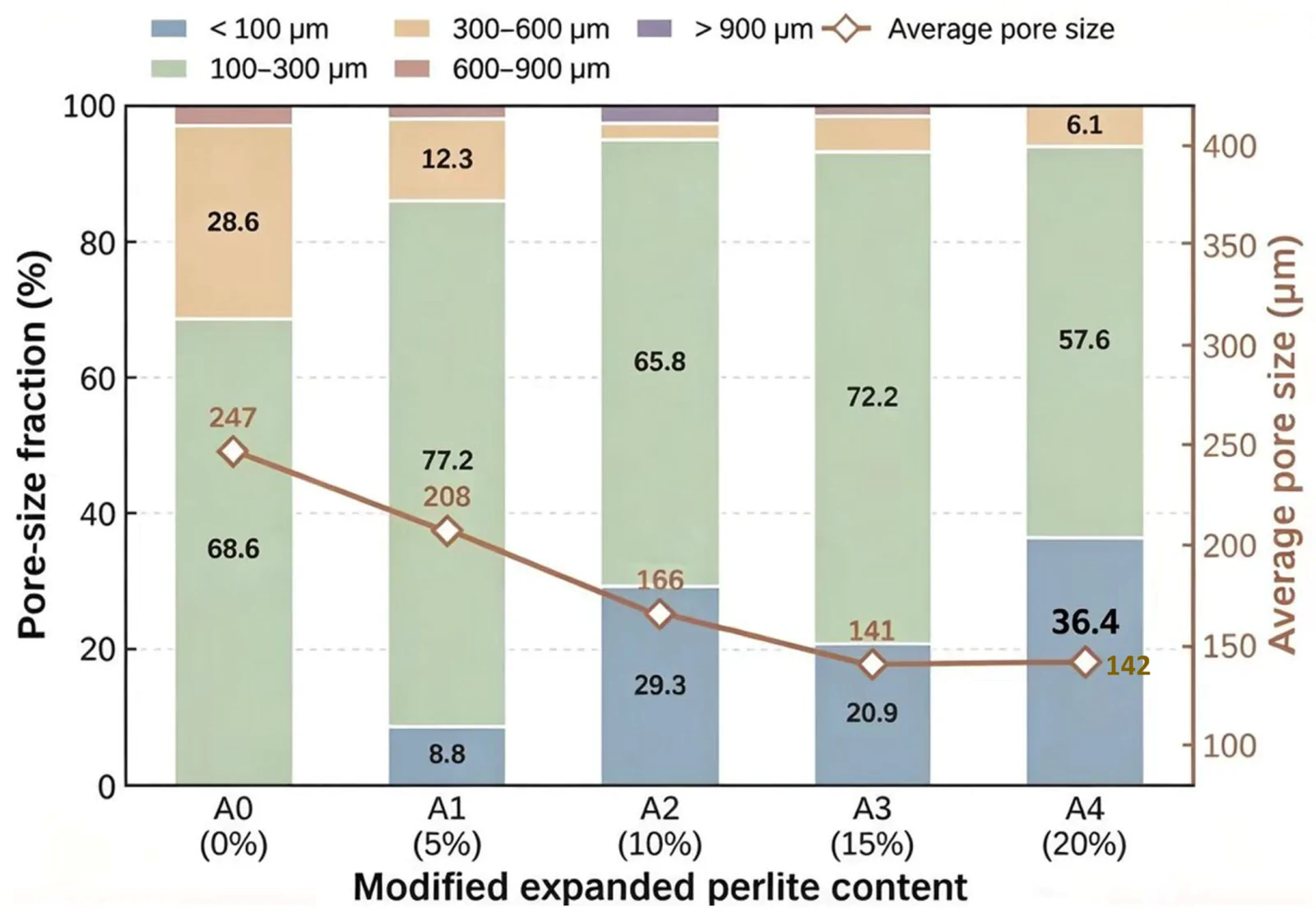
Pore-size distribution and average pore size of foamed concrete with different contents of modified expanded perlite.

Table 5 presents the quantitative analysis of the pore structure obtained with ImageJ, and Figure 9 visualizes the corresponding pore-size distribution together with the evolution of the average pore size. Two optical-microscopy images were acquired for each mixture for pore-size statistics. More than 80 pores were counted for every group by ImageJ software. With increasing modified expanded perlite dosage, the proportion of pores smaller than 300 µm increased continuously, while the proportion of pores larger than 300 µm decreased. Consequently, the average pore size continued to decrease. When the dosage was 0%, the pores were mainly concentrated in the [100, 300) µm range, accounting for 68.57%, with an average pore size of 246.8 µm. At 5%, the proportion of pores smaller than 300 µm increased to 85.96%, and the average pore size decreased to 207.6 µm, a reduction of 16% compared with A0. At 10%, the proportion of pores smaller than 300 µm further increased to 95.12%, with pores smaller than 100 µm accounting for 29.27%, and the average pore size decreased further to 166.0 µm. At 15%, the pore distribution shifted further to the small-pore range, with the proportion below 300 µm remaining at 93.1% and the average pore size decreasing to 140.6 µm. When the dosage reached 20%, the proportion below 300 µm remained at a high level of 93.94%, and the average pore size was 142.5 µm, indicating that the pore structure was still dominated by small pores. As mentioned above, partial inconsistency between λ_1_ from water-absorption-kinetics model and ImageJ-derived average pore-size results is attributed to differences in test principles and surface hydrophobic modification effects.

### 3.7. SEM Observation

**Figure 10:**
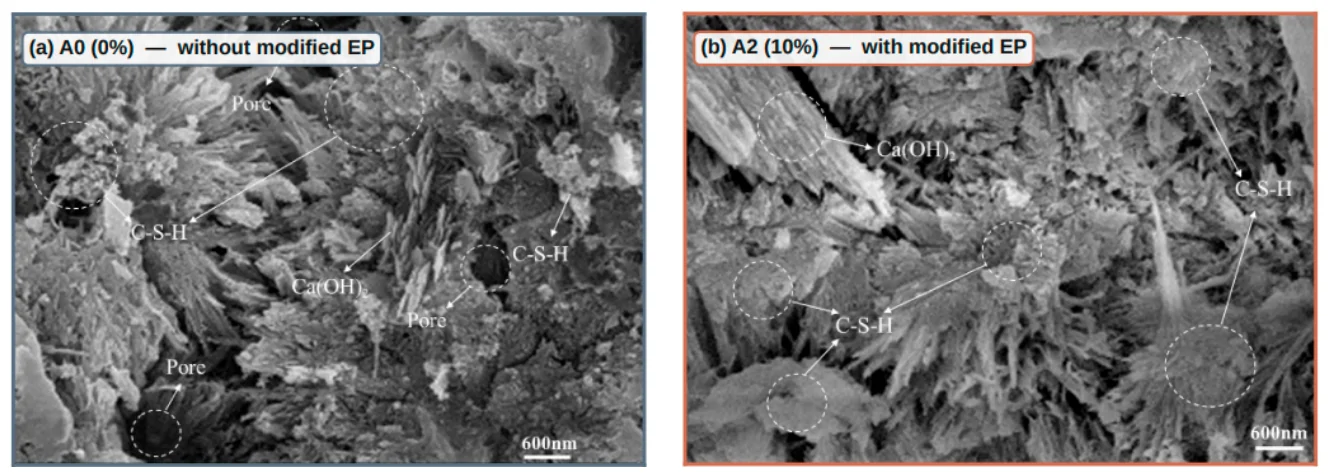
SEM micrographs of foamed concrete: (**a**) without modified expanded perlite (A0); (**b**) with 10% modified expanded perlite (A2). Magnification: 2000×; accelerating voltage: 15 kV.

Figure 10a,b show the SEM micrographs of foamed concrete without perlite and with 10% modified expanded perlite, respectively. The specimen without modified expanded perlite contains many micropores and microcracks, resulting in poor compactness and a relatively small amount of hydration products such as calcium silicate hydrate (C–S–H) gel. After adding 10% modified expanded perlite, a denser microstructure with more hydration products can be observed, and the internal pores and microcracks are markedly reduced, leading to a more uniform structure.

The SEM observations indicate that the addition of modified expanded perlite is beneficial to the development of hydration products. The dense hydrophobic film formed on the perlite surface prevents excessive adsorption of pore solution and stabilizes the local water–binder ratio of matrix. In an alkaline cement environment, expanded perlite may leach silicon- and aluminum-containing components that potentially participate in secondary pozzolanic reactions. However, these hypothesized mechanisms cannot be fully confirmed only by SEM morphology. Further supporting evidence from EDS, XRD or thermal analysis will be required in future research. Together with the particle-filling effect, modified expanded perlite further refines the pore structure, reduces porosity and defect density, and improves microstructural compactness.

### 3.8. Study Limitations

Only hydrophobically modified expanded perlite was adopted in this study. Preliminary tests demonstrated that raw unmodified expanded perlite possesses extremely high water absorption. Direct incorporation of unmodified expanded perlite absorbs massive free water from fresh foamed concrete slurry, destroys foam stability and leads to specimen forming failure. For this practical reason, no parallel groups with unmodified expanded perlite could be prepared. Consequently, it is difficult to completely separate the intrinsic lightweight aggregate effect of expanded perlite and the modification effect induced by sodium methylsilicate treatment. In future work, pre-wetting schemes for raw expanded perlite will be explored to realize comparative tests, so as to distinguish these two effects. Additionally, contact angle, EDS, XRD and thermal analysis were not carried out in the present investigation, which are worthy of further research.

## 4. Conclusions

(1) With the increase in hydrophobically modified expanded perlite dosage, the dry density of foamed concrete decreases firstly and then rises. The measured dry densities range from 985 kg/m^3^ to 1088 kg/m^3^, which are higher than the target value of 800 kg/m^3^ mainly because of foam loss during mixing and casting. The 48 h water absorption is greatly reduced after adding modified EP, decreasing from 20.18% for A0 to 6.11% for A3, while water absorption shows a rebound phenomenon at the 20 wt% replacement level.
(2) The 28 d compressive strength reaches a maximum value of 7.15 MPa at 10 wt% modified EP (A2), which is 111% higher than the control group A0. Excessive dosage over 10 wt% leads to a decrease in compressive strength. Appropriate dosage of modified EP improves freeze–thaw resistance, whereas drying shrinkage continuously deteriorates with increasing EP content.
(3) Modified expanded perlite effectively refines the pore structure and reduces average pore size of foamed concrete. Partial inconsistency exists between the water-absorption-kinetics-calculated λ_1_ and ImageJ-derived geometric average pore size, resulting from different test principles and surface hydrophobic effects. SEM observations show denser microstructure for group A2, but the related hydration mechanisms still need further verification by additional characterization techniques.
(4) Although group A2 (10 wt% modified expanded perlite) achieves the highest compressive strength, its drying shrinkage does not reach the optimal level among all mixtures. Taking compressive strength as primary evaluation criterion and freeze–thaw resistance as auxiliary criterion, 10 wt% modified EP is recommended as the suitable dosage for this study. This recommended dosage does not mean that all tested performance indicators achieve their optimal values under this mixture. Relevant comparative tests and multi-means microscopic analysis will be conducted in future work.

## Figures and Tables

**Table 1:** Physical and mechanical properties of the cement.

Specific Surface Area/m^2^.kg^−1^	Water Requirement for Normal Consistency/%	Soundness by Boiling	Setting Time/min		Compressive Strength/MPa		Flexural Strength/MPa	
361	25.43	Qualified	Initial	Final	3 d	28 d	3 d	28 d
			269	304	23.6	56.73	5.6	8.9

**Table 2:** Absolute-volume calculation of foamed concrete mixtures.

Mixture	Cement Volume/m^3^	EP Volume/m^3^	Water Volume/m^3^	Foam Volume/m^3^	Total Volume/m^3^	Theoretical Fresh Density/kg·m^−3^
A0	0.215	0	0.267	0.570	1.052	923.8
A1	0.204	0.133	0.253	0.570	1.161	825.3
A2	0.194	0.267	0.240	0.570	1.270	743.5
A3	0.183	0.400	0.227	0.570	1.379	674.9
A4	0.172	0.533	0.213	0.570	1.489	616.3

Note: A0–A4 represent mixtures with 0%, 5%, 10%, 15%, and 20% modified expanded perlite, respectively. The volume of foam stabilizer (3.2–4.0 kg per mixture) is negligible and not listed separately.

**Table 3:** Pore characteristic parameters of foamed concrete.

Test Group	Initial Mass/g	15 min/g	1 h/g	24 h/g	48 h/g	*λ*_1_	*α*
A0	1072	1162.20	1207.33	1275.83	1288.30	0.982	0.432
A1	980.67	1005.50	1019.67	1069.33	1083.50	0.477	0.395
A2	1018.17	1040.50	1051.33	1091.67	1107.20	0.467	0.347
A3	1061.33	1079.30	1086.83	1116.83	1126.20	0.499	0.307
A4	1059.50	1083.30	1093.67	1129.17	1138.50	0.565	0.327

**Table 4:** Drying-shrinkage values (×10^−6^) measured at a gauge length of 515 mm.

Test Group	3 d	7 d	14 d	28 d	56 d
A0	798	924	1610	1680	1831
A1	1100	1490	1953	2078	2185
A2	2278	2718	3696	3834	3967
A3	2668	3025	3540	3946	3976
A4	3368	3770	4401	4656	4683

**Table 5:** Pore structure of foamed concrete.

Mixture	Pore-Size Distribution/%					Mean Void Diameter/µm
	<100 μm	[100, 300) μm	[300, 600) μm	[600, 900) μm	>900 μm	
A0	0	68.6	28.6	2.9	0	246.8
A1	8.8	77.2	12.3	1.8	0	207.6
A2	29.3	65.9	2.4	0	2.4	166.0
A3	20.9	72.2	5.6	1.3	0	140.6
A4	36.4	57.6	6.1	0	0	142.5

## Data Availability

The original contributions presented in this study are included in the article. Further inquiries can be directed to the corresponding author.
